# Behavior and possible function of *Arabidopsis* BES1/BZR1 homolog 2 in brassinosteroid signaling

**DOI:** 10.1080/15592324.2022.2084277

**Published:** 2022-06-13

**Authors:** Yui Otani, Mika Kawanishi, Miyu Kamimura, Azusa Sasaki, Yasushi Nakamura, Takako Nakamura, Shigehisa Okamoto

**Affiliations:** a The United Graduate School of Agricultural Sciences, Kagoshima University, Kagoshima, Japan; b Graduate School of Life and Environmental Sciences, Kyoto Prefectural University, Kyoto, Japan; c Department of Japanese Food Culture, Faculty of Letters, Kyoto Prefectural University, Kyoto, Japan

**Keywords:** Brassinosteroid signaling, transcription factor, BES1/BZR1 family, BEH2-regulated genes

## Abstract

Two key transcription factors (TFs) in brassinosteroid (BR) signaling BRASSINOSTEROID INSENSITIVE 1-EMS-SUPPRESSOR 1 (BES1) and BRASSINAZOLE RESISTANT 1 (BZR1), belong to a small family with four BES1/BZR1 homologs (BEH1–4). To date, in contrast to the wealth of knowledge regarding BES1 and BZR1, little is known about BEH1–4. Here, we show that *BEH2* was expressed preferentially in the roots and leaf margins including serrations, which was quite different from another member *BEH4*, and that BRs downregulated *BEH2* through a module containing GSK3-like kinases and BES1/BZR1 TFs, among which BES1, rather than BZR1, contributed to this process. In addition, BEH2 consistently existed in the nucleus, suggesting that its subcellular localization is not under BR-dependent nuclear-cytoplasmic shuttling control. Furthermore, gene ontology analysis on RNA-seq data indicated that BEH2 may be implicated in stress response and photosynthesis. These findings might assist in the future elucidation of the molecular mechanisms underlying BR signaling.

## Introduction

Brassinosteroids (BRs) are polyhydroxylated-steroidal phytohormones categorized as major growth-promoting hormones similar to auxins and gibberellins. BRs play an essential role in growth and development including cell elongation, vascular differentiation, light-dependent alteration of plant architecture, male fertility, and senescence.^1,2^ Moreover, BRs are involved in response to various abiotic and biotic stresses and often confer stress tolerance to plants, which is particularly relevant for economically important crops.^1,3^

In the past two decades, BR signaling has been greatly outlined through molecular genetics, biochemistry, and omics-based approaches.^2,4^ BR signaling in *Arabidopsis* transduces hormonal stimuli from the cell surface to the nucleus through phosphorelay following BR perception by a transmembrane receptor kinase, BRASSINOSTEROID INSENSITIVE 1 (BRI1).^5,6^ BR signals are finally transmitted to a downstream module containing a plant-specific glycogen synthase kinase 3 (GSK3)-like kinase, BRASSINOSTEROID-INSENSITIVE 2 (BIN2) and two similar bHLH transcription factors (TFs), BRI1-EMS-SUPPRESSOR 1 (BES1) and BRASSINAZOLE-RESISTANT 1 (BZR1). In the absence of BRs, BIN2 phosphorylates, and inactivates BES1 and BZR1 through “14-3-3”-mediated cytoplasmic sequestration, proteasomal degradation, and/or reduced affinity to the target promoters.^7–10^ Meanwhile, in the presence of BRs, these TFs are dephosphorylated and activated by the combined action of an inactivated BIN2 and an activated protein phosphatase 2A (PP2A). Consequently, BES1 and BZR1 transcriptionally regulate thousands of genes via either direct binding to the cis-element, BRRE and E-box in their target promoters or modulating gene regulation by their binding partner proteins,^11–13^ which partly accounts for the pleiotropic effects of BR hormones.

The several steps involved in BR signaling are redundantly controlled by different members of protein families, such as BR receptors (BRI1, BRL1, and BRL3), BR co-receptors (SERK1, SERK3/BAK1, SERK4, and SERK5), and GSK3-like kinases (BIN2, ASKι, and ASKζ).^10, 14, 15^ Likewise, BES1 and BZR1 TFs belong to a small family, which includes the other four members: BES1/BZR1 homolog 1–4 (BEH1–4) and can jointly control BR-mediated gene expression. Functional redundancy undoubtedly gives the robustness to BR pathway enough not to collapse even if some of the components are impaired, as a fail-safe design of aircrafts. However, this elaborate system often causes the difficulty to access the precise roles of each member and their relationship among the family. So far, among all family members, most research attention has been paid on BES1 and BZR1.^11^ Thereby, characterization of BEH1–4 has been left behind although the related publication is gradually increasing in recent years. For instance, BEH2–4 proteins are proposed to be involved in strigolactone hormone signaling because they interact with MAX2, its major signaling component.^16^ Furthermore, BEH1–4 may be involved in stomata formation because they are transcriptionally controlled by SPEECHLESS, a key TF in stomata differentiation.^17^ Along this line, we previously profiled the expression of *BEH1–4* in *Arabidopsis* and found that *BEH2* and *BEH1* (to a lesser extent) were downregulated by brassinolide (BL), an active BR, suggesting a close association with BR function.^18^ Furthermore, BEH2 is phosphorylated by ASKθ, a newly-found GSK3-like kinase acting in BR signaling.^19^ Therefore, we focused on and characterized BEH2 to advance our knowledge on BR-mediated gene regulation.

In this study, we disclosed that *BEH2* was differently expressed from other family members in a spatiotemporal manner, and that it was transcriptionally downregulated by BL through a canonical BR pathway. In addition, BEH2 was consistently localized in the nucleus, even if its phosphorylation status changed with BR levels. Furthermore, RNA-seq linked gene enrichment analysis showed an overrepresentation of terms related to stress response and photosynthesis with *BEH2* overexpression.

## Materials and methods

### Chemicals

Chemicals were purchased from FUJIFILM Wako Pure Chemical Corporation (Osaka, Japan) unless otherwise noted. Brassinolide (BL) was purchased from Brassino Co., Ltd. (Toyama, Japan). A BR biosynthesis inhibitor, brassinazole (Brz), and a specific inhibitor of plant GSK3-like kinases, bikinin were kindly provided from Drs T. Asami (Tokyo University) and K. Hayashi (Okayama University of Science), respectively.

### Plants and growth conditions

All plants used in this study shared the genetic background of *Arabidopsis thaliana* ecotype Columbia (Col-0). Gain of function-mutants of *BES1* and *BZR1* (*bes1-D, bzr1-1D*) were provided by Dr J. Chory (The Salk Institute, La. Jolla, CA). A *bes1-D* mutant was backcrossed three times with Columbia wild type (WT) to change its background from En2 to Col-0. T-DNA insertion mutants, *bes1* (SALK_098634 and SALK_091133) and *bzr1* (GK-857E04), were obtained from the Arabidopsis Biological Resource Center (Ohio State University). Three transgenic *Arabidopsis* named *“BEH2::GUS,” “35S::BEH2:GFP”* and *“35S::BZR1:GFP”* were generated in our laboratory. Media, seed sterilization, and growth conditions followed those described in our previous report.^18^

### Plasmid construction and Agrobacterium-mediated transformation

A transcriptional fusion of the *GUS* gene driven by the *BEH2* promoter (*BEH2::GUS*) was constructed as with *BEH4::GUS*.^18^ Meanwhile, translational fusions of either *BEH2* or *BZR1* with *GFP* driven by 35S promoter (*35S::BEH2:GFP* and *35S::BZR1:GFP*) were constructed as described below. After checking sequence accuracy, PCR-cloned cDNA harboring their entire coding sequence but not stop codon were fused in-frame to *sGFP* on a pTH2 plasmid, kindly provided from Dr Y. Niwa (Shizuoka University).^20^ The resulting translational fusions were then moved into the T-DNA region of a binary vector pCAMBIA1300.^21^ *Agrobacterium tumefaciens* carrying the above fusions were subjected to the floral-dip method for *Arabidopsis* transformation.^22^

### Pharmacological analysis

Pharmacological treatment was conducted by culturing seedlings in a half-strength (1/2) Murashige and Skoog (MS) media containing the following chemicals: BL, Brz, and bikinin were dissolved in 100% dimethyl sulfoxide (DMSO) and added into the media at the specified concentrations; final DMSO concentration should not exceed 0.1%.

### Biolistic bombardment

Biolistic bombardment was employed to transiently introduce either *35S::BEH2:GFP, 35S::BZR1:GFP* or *35S::GFP* on the pTH2 to onion epidermal tissues according to Sanford et al.^23^ Plasmid DNA (0.8 µg) absorbed on gold particles (0.5 mg) was shot into the tissues by Biolistic PDS-1000/He Particle Delivery System (Bio-Rad, Richmond, CA). After bombardment, the onion tissues were cultured for 1 day in darkness on 1/2 MS plate with or without the chemicals described above.

### Reporter assay

Histochemical staining and biochemical assay for GUS activity were performed according to Otani et al.^18^ and Yoshimitsu et al.^24^ respectively. GFP fluorescence in transgenic *Arabidopsis* and onion epidermal tissues was imaged using a fluorescence microscope: BZ-9000 with an optical filter: OP-66835 BZ filter GFP (Keyence Corporation, Osaka, Japan). Nuclear counterstain was performed using 4’,6-diamidino-2-phenylindole dihydrochloride (DAPI) according to the supplier’s instruction (Dojindo Lab, Kumamoto, Japan).

### Semi-quantitative reverse transcription PCR (sqRT-PCR)

Semi-quantitative reverse transcription PCR was conducted as described by Otani et al.^18^ Detailed primer information is presented in Supplementary Table 1.

### RNA-seq, gene ontology, and Kyoto encyclopedia of genes and genomes analyses

Total RNA was extracted from 14-day-old WT and *35S::BEH2:GFP* seedlings incubated for 4 h in 1/2 MS liquid medium with or without 0.1 μM BL. After validation of RNA integrity by 1.6% agarose gel electrophoresis and BL effectiveness on marker gene expression by sqRT-PCR, the four RNA samples were shipped to Hangzhou Veritas Genetics Medical Institute Co. Ltd. (Hangzhou, China), where RNA-seq was performed using the Illumina NovaSeq 6000 Sequencing System (150 bp paired-end reads; 3 G). To obtain count data, poor-quality reads were first filtered out using Trim Galore (ver0.6.4) software with default parameters^25^ after whole read quality-checking by FastQC (ver0.11.8).^26^ Next, the high-quality reads were mapped to the reference genome of *Arabidopsis* TAIR10 using STAR (ver2.7.4a) with default parameters.^27^ Then, the read count for each gene, defined by “gene symbol” (Ensembl v27), was calculated using featureCounts (v.1.6.4).^28^ Expression of all analyzable genes was normalized by aligning the total read count in each of the four RNA-seq groups to a million (CPM, count per million). Following TMM (Trimmed mean of M values) normalization of the count data, gene expression was analyzed in one-to-one comparison among four RNA-seq groups using edge R (ver 3.22.3),^29^ and differentially expressed genes (DEGs) were defined as those with a fold change either >2 or <0.5. Gene ontology (GO) and Kyoto encyclopedia of genes and genomes (KEGG) analyses were performed using the g:profiler online tool (version e105_eg52_p16_e84549f) with g:SCS multiple testing correction method applying a significance threshold of 0.05.^30^

## Results

### BEH2 gene: tissue specific expression and responsiveness to BR

**Figure 1.**
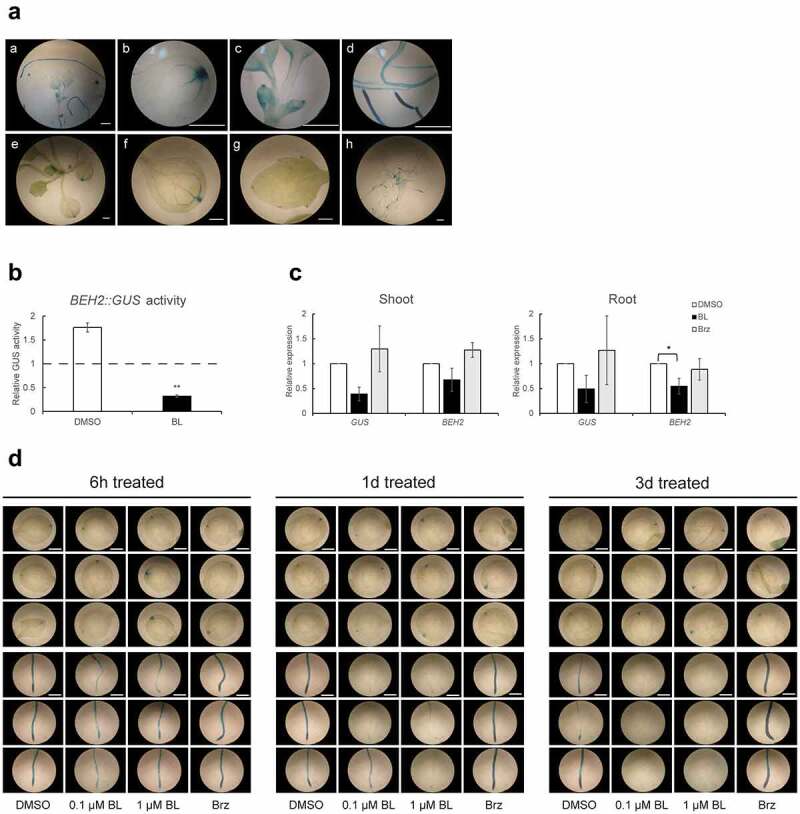
Responsiveness of *BEH2* expression to different BR levels.

Histochemical GUS staining was performed to determine the precise sites of *BEH2* expression at the tissue level, although our previous work showed that it expressed in various organs.^18^ GUS staining was detected in almost all organs except hypocotyl in 1- and 2-week-old *BEH2::GUS* plants, among which the staining was more obvious in the roots than in other organs (Figure 1a). Additionally, the staining was observed in confined regions of each organ. For instance, the meristematic- and elongation zones in the roots tended to be more intensely stained than the differentiation zone. Cotyledons were stained at the leaf blade tip with continuously linked veins (Figure 1a(b, f)). Young developing rosette leaves (1-week-old) were stained in the whole blade, however, as they developed, the staining became restricted to the serrations (Figure 1a(g)) and the marginal regions at the basal part of the leaves (Figure 1a(c)).

*BEH2::GUS* plants were used for all experiments in this figure. (**a**) 1-week-old (a-d) and 2-week-old (e-h) plants were subjected to histochemical GUS staining. Photographs represent the whole seedlings (a, e), cotyledons (b, f), true leaves (c, g), and roots (d, h). (**b**) Following 3 days treatment of 1 µM BL or 0.1% DMSO, 17-day-old plants were subjected to biochemical GUS assay. The activity is shown as a relative value (means ± S.D) to that of the initial control (time zero) in the graph. (**c**) Following 24 h treatment of 1 µM BL, 5 µM Brz or 0.1% DMSO, RNA prepared from the roots and shoots of 15-day-old plants were subjected to sqRT-PCR. In the graphs, relative expression values (means ± S.D) to that of the DMSO control calculated after normalization to *ACT2* are presented. (**d**) 5-day-old seedlings were treated for the defined periods with 0.1- and 1-µM BL or 5 µM Brz and then subjected to histochemical staining. Statistical analysis was performed by Student’s t test (**p < .01) for (**b**) and by ANOVA with Dunnett’s test (*p < .05) for (**c**). White bars in photographs (**a**, **d**) represent 1 mm.

*BEH2* expression is reportedly downregulated by BRs at the mRNA level.^12,13,18^ Therefore, to elucidate if BRs transcriptionally regulate *BEH2* expression, we examined the effect of BL on the GUS activity driven by *BEH2* promoter. As shown in Figure 1b, the activity in *BEH2::GUS* plants (17-day-old) treated with 1 µM BL for 3 days was nearly one-fifth of that in the DMSO control and less than a half of that in the initial control (14-day-old). Using sqRT-PCR, we next examined the expression of the endogenous *BEH2* gene and transgenic *BEH2::GUS* gene in the same transgenic plant to evaluate if mRNAs from the two genes fluctuate with BR levels, and which part, shoots, or roots, are involved in BR-triggered *BEH2* downregulation. As shown in Figure 1c, BL tended to lower mRNA levels of both genes compared with the DMSO control, whereas Brz did not have an influence or even slightly induced their mRNA. Additionally, the responsiveness to BR levels was quite similar between shoots and roots. Then, we histochemically examined if *BEH2::GUS* responded to BR levels at the cotyledonous stage (5-day-old). As shown in Figure 1d, BL and Brz clearly reduced and increased staining in the roots, respectively, at 1 day and 3 days of chemical treatment. In contrast, they did not influence much GUS staining in cotyledons. Altogether, these results suggest that BRs downregulate *BEH2* transcriptionally, and that this regulation occurs in both shoots and roots.

### *BR-triggered* *BEH2* *downregulation follows the canonical signaling pathway*

**Figure 2.**
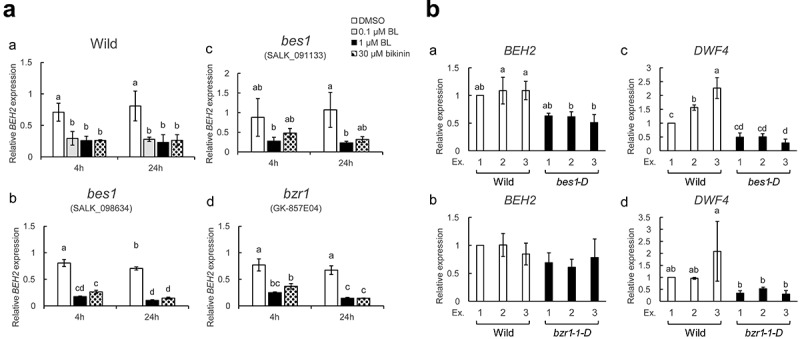
Effects of bikinin and *bes1*/*bzr1* mutations on *BEH2* expression.

BR transcriptionally regulates thousands of genes through inactivation of BIN2 kinase, a major negative regulator of BR signaling.^31^ Therefore, we examined if BIN2 and its family members participate in BR-mediated *BEH2* repression using bikinin, a GSK3-like kinase inhibitor.^32^ As shown in Figure 2a(a), *BEH2* mRNA decreased to less than a half of the DMSO control by administration of 30 µM bikinin for 4 and 24 h, similar to BL treatment. We also conducted the same analysis using T-DNA insertion (loss of function) mutants of *BES1* (SALK_098634 and SALK_091133) and *BZR1* (GK-857E04) to determine to what extent their impairment influences *BEH2* regulation. *BEH2* mRNA fluctuated with bikinin and BL in the three mutants, similar to what occurred in the WT (Figure 2a (b–d)). Therefore, we examined *BEH2* mRNA levels in their dominant (gain of function) mutants, *bes1-D* and *bzr1-1D*. As shown in Figure 2b (a, b), the mRNA level in *bes1-D* was about half that in WT, while no noticeable decrease was observed in *bzr1-1D*. In contrast, *DWF4* mRNA significantly decreased in both mutants compared to WT (Figure 2b (c, d)), as expected from the fact that BES1 and BZR1 downregulate *DWF4*.^33,34^ These results indicate that BRs downregulate *BEH2* through GSK3-like kinase inactivation^32^ and activation of BES1/BZR1 family members, among which BES1, rather than BZR1, contributes to this process.

(**a**) 14-day-old *Arabidopsis* WT (a; Col-0) and T-DNA insertion mutants for *bes1*(b; SALK_098634, c; SALK_091133) and *bzr1* (d; GK-857E04) were treated with either BL or bikinin for 4 and 24 h, and subjected to sqRT-PCR for evaluating *BEH2* mRNA level. (**b**) 14-day-old seedlings (three seedlings of each mutant) of the dominant mutants *bes1-D* and *bzr1-1D* were directly subjected to sqRT-PCR for evaluating mRNA levels of *BEH2* (a, b) and *DWF4* (c, d). Presentation styles in the graphs follow those in Figure 1. Statistical analysis was performed by ANOVA with Tukey’s test (p < .05).

### BEH2 protein: subcellular localization

**Figure 3.**
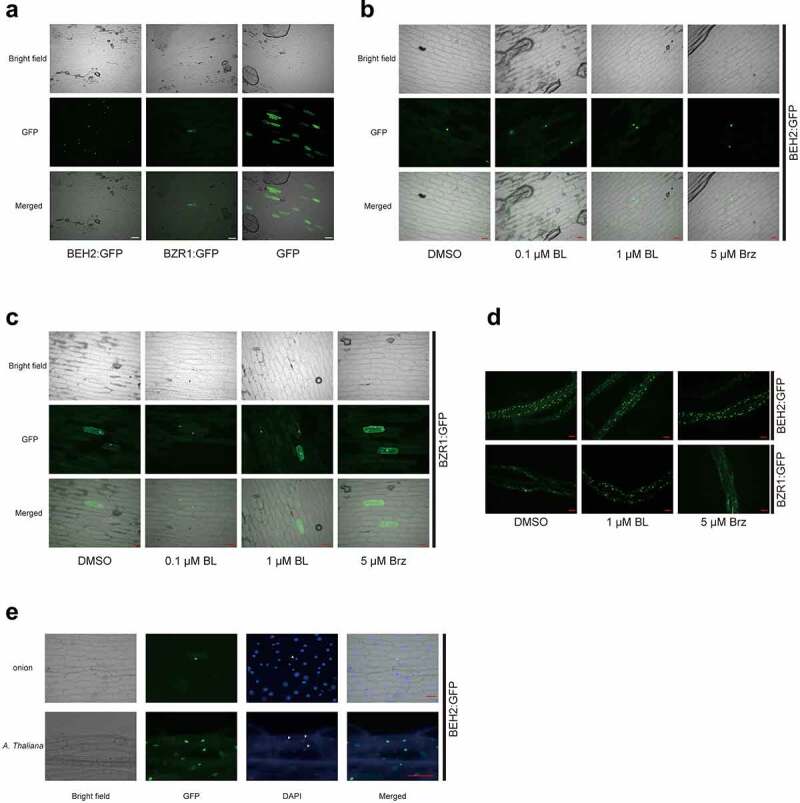
Subcellular localization of BEH2 protein.

BRs modulate the subcellular localization of BZR1 and BES1 for BR signaling efficacy.^35,36^ Therefore, we observed GFP fluorescence from BEH2:GFP fusion proteins to elucidate if a nuclear-cytoplasmic shuttling mechanism modulates BEH2 activity. Interestingly, when cultured without chemicals, GFP fluorescence was observed mostly in the nuclei of onion epidermal cells transiently expressing *35S::BEH2:GFP* gene (Figure 3a), which was confirmed by co-localization of the fluorescence signals of GFP and DAPI (Figure 3e). In contrast, the fluorescence from *35S::BZR1:GFP* and *35S::GFP* was detected in both cytoplasm and nucleus. We then treated onion epidermal tissues with either BL or Brz. The BEH2:GFP fluorescence consistently existed in the nucleus, regardless of chemical used (Figure 3b). In contrast, BZR1:GFP fluorescence was observed in both fractions, but its strength changed due to the chemicals; the fluorescence was more intense in the nucleus than in the cytoplasm when treated with BL, and vice versa with Brz (Figure 3c).

Plasmid DNA carrying either *35S::BEH2:GFP, 35S::BZR1:GFP* or *35S::GFP* was biolistically bombarded into onion epidermal tissues, and the tissues were cultured in dark for 1 day on 1/2 MS solidified medium without (**a**) or with chemicals (**b** and **c**). Seven-day-old seedlings of *Arabidopsis* harboring *35S::BEH2:GFP* or *35S::BZR1:GFP* were cultured in continuous light for 1 day in 1/2 MS liquid medium containing BL or Brz (**d**); next, the roots were excised for microscopic observation. The onion peels and *Arabidopsis* roots producing BEH2:GFP proteins were stained with DAPI to determine the position of nucleus (**e**). Red and white scalebars represent 100 and 300 µm, respectively. White arrowheads in (**e**) indicate the position of DAPI signal in *GFP* expressing cells.

As BEH2:GFP fluorescence was consistently detected in the nucleus of onion cells, we subjected the transgenic *Arabidopsis* harboring *35S::BEH2:GFP* to the same observation. As shown in Figure 3d and 3e, BEH2:GFP fluorescence was detected in the nuclei of root cells in the seedlings; the nuclear localization of BEH2 was hardly affected by BL and Brz, while BZR1:GFP fluorescence was detected in both nucleus and cytoplasm and changed similarly to onion cells. Together, the results indicate that, unlike BZR1, BEH2 proteins are consistently localized in the nucleus and not modulated by a nuclear-cytoplasmic shuttling mechanism depending on BR levels.

### Putative BEH2-regulated genes and BEH2ʹs involvement in BR signaling

**Figure 4.**
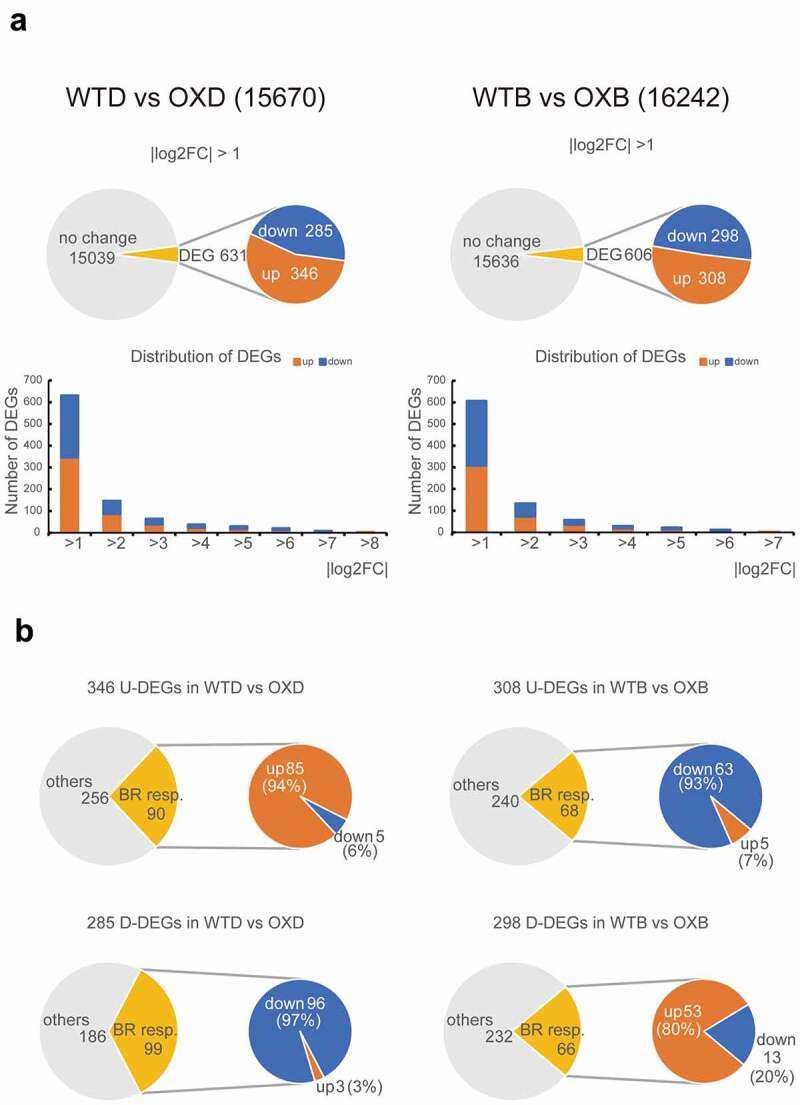
Transcriptome profiling of BEH2-regulated genes.

For mining BEH2-regulated genes, RNA-seq was performed to evaluate transcriptomic differences between the parental *Arabidopsis* WT and the *BEH2* overexpressing (BEH2OX) plant in which *BEH2* gene was expressed six times higher than in WT (Supplementary Figure 1). As shown in Figure 4a, 15670 expressed genes were used for comparison of WT and BEH2OX whose seedlings were grown in the absence of BL (under 0.1% DMSO; WTD vs OXD). Among them, 631 DEGs with |log2FC| >1, comprising 346 upregulated genes (U-DEGs in WTD vs OXD) and 285 downregulated genes (D-DEGs in WTD vs OXD) were found. Similarly, 606 DEGs with |log2FC| >1, comprising 308 upregulated genes (U-DEGs in WTB vs OXB) and 298 downregulated genes (D-DEGs in WTB vs OXB) were found from 16242 genes used in WT and BEH2OX comparison, whose seedlings were grown in the presence of BL. DEGs with |log2FC| ≤2 account for approximately 75% of their total number, irrespective of BL administration (Figure 4a). Furthermore, 106 genes were common between DEGs obtained with (606) and without (631) BL treatment, indicating that *BEH2* overexpression affected the expression of ≥1131 genes. BES1 and BZR1 have already been demonstrated to have 1609 and 3410 direct target genes, respectively, by ChIP-chip analyses.^12,13^ Therefore, we next examined to what extent BEH2OX-mediated DEGs (1131) were common to them and found 41 and 134 were included in the target gene pools of BES1 and BZR1, respectively, among which 18 were co-targeted by BES1 and BZR1 (Supplementary Figure 2).

(**a**) Pie charts indicating the proportion of differentially expressed genes (DEGs) with |log2FC| >1 among the total genes (15,670 and 16,242) used for comparison between WT and BEH2OX grown in the absence (WTD vs OXD) or presence of BL (WTB vs OXB). Bar graphs presenting the ratio of DEGs with different |log2FC| values. Note that approximately three-quarters of DEGs have |log2FC| ≤2. “Up” and “down” mean upregulated and downregulated genes in BEH2OX seedlings, respectively. (**b**) Pie charts presenting the proportion of BR-responsive genes in upregulated DEGs (U-DEGs) and downregulated DEGs (D-DEGs) in individual comparisons (WTD vs OXD, WTB vs OXB).

We then compared BEH2OX-mediated DEGs and BL-mediated DEGs with |log2FC| >1 [U-DEGs (406) and D-DEGs (446) in WTD vs WTB; Supplementary Figure 3] to evaluate if and to what extent BRs regulated BEH2OX-mediated DEGs (Figure 4b). Among the 346 U-DEGs in WTD vs OXD, 90 genes were BL responsive and included 85 upregulated and 5 downregulated genes; among the 285 D-DEGs in WTD vs OXD, 99 BL responsive genes, 96 downregulated and 3 upregulated, were found. Similarly, 68 BL responsive genes were found in the 308 U-DEGs in WTB vs OXB, including 63 downregulated and 5 upregulated genes, while in the 298 D-DEGs in WTB vs OXB, there were 66 BL responsive genes, comprising 53 upregulated and 13 downregulated genes. This result implies that 20%–35% of BEH2OX-mediated DEGs are BR-regulated [U-DEGs (90/346) and D-DEGs (99/285) in WTD vs OXD; U-DEGs (68/308) and D-DEGs (66/298) in WTB vs OXB], and that BR administration and *BEH2* overexpression regulate these genes in either the same- or opposite direction depending on BR level, which was also confirmed in a heatmap analysis using the expression ratio (logFC) (Supplementary Figure 4).

**Figure 5.**
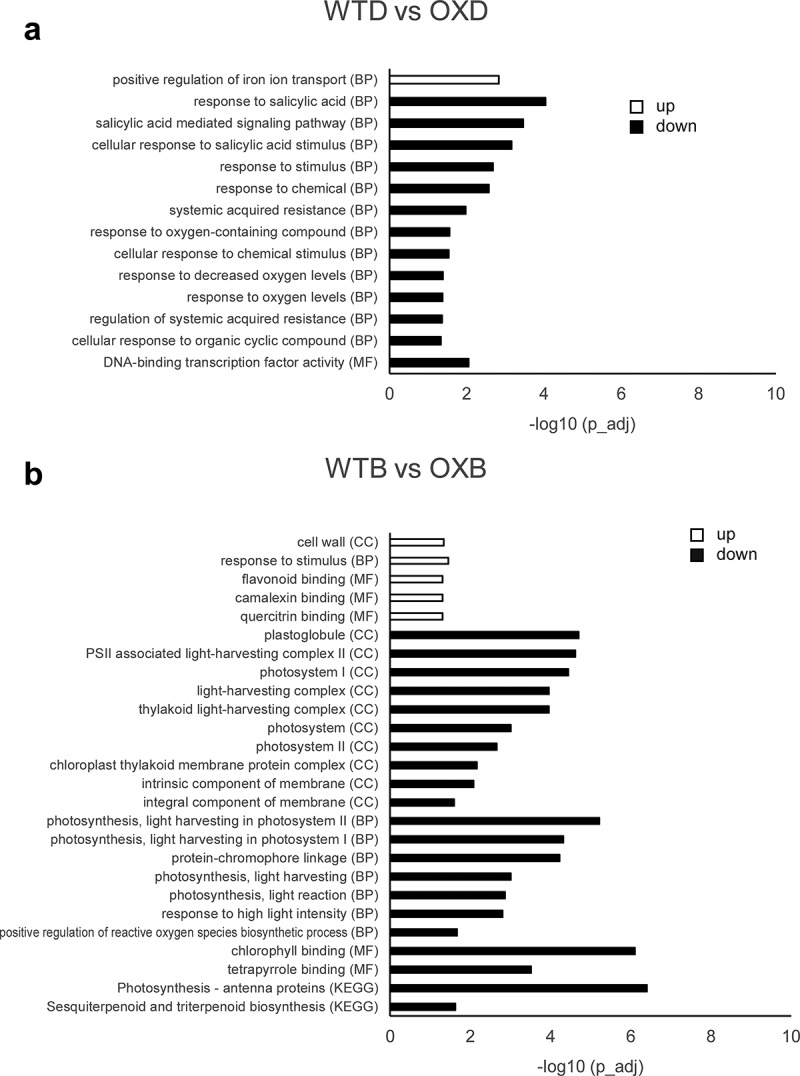
GO and KEGG enrichment analysis of BEH2-regulated genes.

To further address the potential BEH2 functions, GO and KEGG pathway analyses were performed with the four sets of DEGs (U-DEGs and D-DEGs in both WTD vs OXD and WTB vs OXB). Only one GO:Biological Processes (BP) term “positive regulation of iron ion transport” was found in the U-DEGs of WTD vs OXD, while 12 GO:BP and one GO:Molecular Functions (MF) terms were enriched in the D-DEGs of WTD vs OXD (Figure 5a). The 12 GO:BP terms were strongly associated with stress and defense responses, like chemical stress, salicylic acid-related process, systemic acquired resistance, and oxidative stress (Table 1). Furthermore, all genes in each GO:BP term were included in “response to stimulus.” Twenty-one genes among 33 in the GO:MF “DNA-binding transcription factor activity” were found among the stress-related GO:BP terms, suggesting a close link between BEH2 with response to stimuli. Meanwhile, quite different GO terms were overrepresented under the influence of BL (Figure 5b). Five GO terms (1 GO:CC, 1 GO:BP and 3 GO:MF) related to cell wall, stress response, and chemical binding were enriched in the U-DEGs of WTB vs OXB. In contrast, 19 GO terms (10 GO:CC, 7 GO:BP and 2 GO:MF) as well as two KEGG pathways were overrepresented in the D-DEGs of WTB vs OXB. Interestingly, all enriched GO terms and KEGG pathways were associated with chloroplasts, which is likely related to the photochemical reactions required for photosynthesis and/or photo-stress responses (Figure 5b, Table 2). Altogether, our GO and KEGG enrichment analyses imply that BEH2 may be involved in stress and defense responses as well as photosynthesis-related processes.

Enrichment analysis conducted separately for U-DEGs (open bar) and D-DEGs (closed bar) in individual comparisons (**a**, WTD vs OXD; **b**, WTB vs OXB). The terms were defined by adjusted p-value < 0.05 after multiple testing corrections using the default g:SCS algorithm. In the graphs, the adjusted p-values (p_adj) of each term are shown in negative log 10 scale. GO, Gene Ontology; KEGG, Kyoto Encyclopedia of Genes and Genomes; BP, Biological Processes; CC, Cellular Components; MF, Molecular Functions.

**Table 1.** Summary of enrichment analysis for D-DEGs in WTD vs OXD.

source	category	term	gene
GO:BP	chemical stress	4	56
GO:BP	salicylic acid related process	3	12
GO:BP	systemic acquired resistance	2	7
GO:BP	oxidative stress	2	11
GO:BP	stimulus	1	94
GO:MF	transcription	1	33

**Table 2.** Summary of enrichment analysis for D-DEGs in WTB vs OXB.

source	category	term	gene
GO:CC	photosynthesis	7	8
GO:CC	membrane	2	99
GO:CC	plastoglobule	1	9
GO:BP	photosynthesis	5	11
GO:BP	photo-stress	2	7
GO:MF	chlorophyll binding	1	8
GO:MF	tetrapyrrole binding	1	18
KEGG	photosynthesis – antenna proteins	1	6
KEGG	sesquiterpenoid and triterpenoid biosynthesis	1	3

## Discussion

So far, little is known about BEH1–4, the other BES1/BZR1 family members in contrast to the wealth of knowledge for the two central TFs, BES1, and BZR1 in BR signaling. Therefore, in this study, we focused on BEH2 to advance our knowledge on BR-mediated gene regulation.

Our expression analysis unveiled the detailed patterns of *BEH2* expression at the tissue level (Figure 1a): *BEH2* was expressed in the confined regions in each organ; its expression was more intense in roots than shoots, suggesting that roots are likely the primary site of *BEH2* expression; *BEH2* expression pattern was quite different from those of *BES1* and *BEH4* that are ubiquitously expressed^18,33,36^ and from *BEH3* that is expressed in leaves and petioles, especially their vascular tissues.^37,38^ Together, these results indicate that each BES1/BZR1 family member might play distinct physiological roles by differently modulating their expression, despite their common roles based on their structural similarity.

*BEH2, BEH1*, and *BES1* mRNA levels reportedly fluctuate in a BR-dependent manner; BL reduced *BEH1* and – particularly–*BEH2*, and induced *BES1*.^13,18^ Therefore, we examined if BL downregulates *BEH2* transcriptionally. BL significantly reduced GUS activity in *BEH2::GUS* seedlings (Figure 1b) and histochemical staining further proved that this reaction surely occurred in roots but not much in shoots at the cotyledonous stage (Figure 1d). However, sqRT-PCR showed that BL reduced endogenous *BEH2* mRNA and introduced *GUS* mRNA in shoots and roots of 14-day-old plants (Figure 1c). Together, these results indicate that BR downregulates *BEH2* via transcriptional control although we cannot exclude the possibility of posttranscriptional control. In addition, this downregulation should occur in the whole plant body (Figure 1c). Given that BL transcriptionally downregulates *BEH2*, we then examined if *Arabidopsis* GSK3/Shaggy-like kinases (ASKs) including BIN2 as well as BES1- and BZR1-TFs are involved in this regulation. Accordingly, bikinin decreased *BEH2* mRNA similar to BL in WT and even in *BES1* and *BZR1* loss-of-function mutants (Figure 2a), suggesting a close connection of ASKs with BR-triggered *BEH2* down regulation, and also the functional redundancy in the BES1/BZR1 family. The *BEH2* mRNA level was reduced in *bes1-D* but not so much in *bzr1-1D*, compared with WT (Figure 2b (a, b)), suggesting that BES1 was more associated with this response than BZR1. These results indicate that BR downregulates *BEH2* in a canonical BR pathway. Thus far, seven of 10 ASKs reportedly function as negative regulators in BR signaling: BIN2, ASKι, and ASKζ (group II), ASKα, ASKγ, ASKε (group I), and ASKθ (group III).^32^ Therefore, when BR level is elevated or bikinin is added, the inactivated ASKs in combination with PP2A probably dephosphorylate and activate BES1/BZR1 family TFs, ultimately leading to *BEH2* downregulation. The high functional redundancy caused by paralogs, i.e., ASK- and BES1/BZR1- family proteins, can greatly contribute to protecting cellular machinery from their genetic impairments, indispensable for plant survival; however, this often cause research difficulties. Thus, a next challenge would be to overcome these problems and determine which ASKs and BES1/BZR1 family TFs regulate *BEH2*.

The actions of BES1 and BZR1 are mostly modulated at the protein level, i.e., proteasome-mediated degradation, nuclear-cytoplasmic shuttling, and change in DNA-binding affinity, all occurring in a phosphorylation-dependent manner.^7,8,10,35,36,39^ BEH2 is reportedly dephosphorylated by BR.^40^ Furthermore, ASKθ acting in BR signaling binds and phosphorylates BEH2 protein *in vitro* and *in vivo*.^19^ Therefore, we investigated BEH2 phosphorylation and subcellular localization using *35S::BEH2:GFP* plants. Immunoblot analysis showed that the phosphorylation status of BEH2:GFP fusion proteins changes conversely by BL (toward the dephosphorylated state) and Brz (toward the phosphorylated state) (Supplementary Figure 5), as in the study of Yin et al.^40^ However, our microscopic observation showed that BEH2:GFP fluorescence was always detected in the nucleus, regardless of the chemical applied (Figure 3). This observation indicates that BEH2 is consistently localized in the nucleus independent of BR content, and that its action is not likely modulated by nuclear-cytoplasmic shuttling. This finding is of interest because BEH2 has a putative 14-3-3 recognition site and a putative bipartite nuclear localization sequence.^41,42^ The predicted 14-3-3 site (PLRISN[S]APVT) in BEH2 is similar to that of BES1 (PLRISN[S]APVT) and BZR1 (SLRISN[S]CPVT), required for their cytoplasmic retention.^9,35^ What caused the difference of BEH2 behaviors regarding intracellular movement from BES1 and BZR1? Ryu et al.^35^ described the existence of another domain including S-130 and S-134 residues in BZR1, which is targeted by BIN2 and required for BZR1ʹs nuclear export. We found that three residues either in or flanking this domain differed (Q-128 in BZR1 vs H-121 in BEH2; V-129 in BZR1 vs G-122 in BEH2; S-132 in BZR1 vs V-125 in BEH2), although the two corresponding serine residues were conserved in BEH2. Among them, the two replacements resulted in a substantial alteration in amino acid properties; thus, the difference in this domain may cause the constitutive nuclear localization of BEH2. This question should be addressed to further characterize BEH2.

Global gene expression analysis is a powerful tool to approach gene function. RNA-seq provided information about BEH2-regulated genes. First, we uncovered that ≥1131 genes were differentially expressed in BEH2OX plants compared to WT (Figure 4a). This number was lower than the DEGs detected in *bes1-D* mutant (4194) and in *bzr1-1D;bri1-116* double mutant (6742),^12,13^ suggesting that BEH2 regulates a smaller number of genes compared to the two central TFs although we have to carefully evaluate their ability with deeply considering the difference in experimental conditions such as plant age, growth conditions, and analytical methods for gene expression. However, we can still claim that BEH2 can potentially regulate over a thousand genes directly or indirectly. Second, we found that around a quarter of BEH2OX-mediated DEGs are BL responsive in WT (Figure 4b), strongly suggesting its involvement in BR signaling, which agrees with previous reports.^18,19,40^ However, over 65% of BEH2OX-mediated DEGs were not apparently affected by BL in WT and a similar situation is also observed in case of BES1 and BZR1, i.e., only 404 of 1609 BES1 target genes are regulated by BRs and/or in *bes1-D* mutant;^13^ 2457 of 3410 BZR1 targets are out of BR control.^12^ Therefore, these results may imply existence of the unknown gene regulatory mechanism by BES1/BZR1 family TFs although Yu et al.^13^ discussed in their report that BR regulation of these genes may not be detectable under tested conditions in WT but their regulations by BES1 are magnified in *bes1-D*. Third, we disclosed that the direct targets of BES1 and BZR1 were only a small portion (157 in total) of BEH2OX-mediated DEGs (1131) (Supplementary Figure 2). This result was contrary to our expectation, because of the functional redundancy of BES1/BZR1 family TFs evidenced by the facts that their recessive mutants with phenotype have not been isolated by forward genetics.^43^ There are some possible explanations for our finding. For instance, BEH2 may have other physiological roles different from BES1 and BZR1, or BEH2 may behave redundantly with BES1 and BZR1 at the point of physiological output, even if their primary targets significantly differ. In any case, we must wait for further characterization of each BES1/BZR1 family member.

Our GO and KEGG results imply that BEH2 is implicated in two physiological processes: “stress response” and “photosynthesis” (Figure 5, Tables 1 and 2). It is noteworthy to recognize that “stress response” and “photosynthesis” emerged under the growth conditions without and with BL, respectively; however, the factors causing this difference remain unknown. Hitherto, numerous articles have reported a close relationship of BRs with stress response and photosynthesis. However, the role of BR in stress responses has not been determined yet, although BRs reportedly influence either positively or negatively on plant responses to various stresses.^44–47^ Meanwhile, BRs are known to positively contribute to photosynthetic assimilation by enhancing chlorophyll biosynthesis, maintaining photosystem II efficacy, and elevating photosynthetic carbon fixations.^48–50^ Thus, our finding might help further elucidate the underlying mechanisms of BR-mediated stress response and photosynthesis by providing BEH2 TF as a new research target.

## Supplementary Material

Supplemental Material

## Data Availability

All sequence data have been submitted to the DDBJ databases under accession number DRA014010.
