# Supplementary material for: Behavior and possible function of Arabidopsis BES1/BZR1 homolog 2 in brassinosteroid signaling
Source: Plant Signal Behav. 2022 Jun 13;17(1):2084277. doi: 10.1080/15592324.2022.2084277 (PMC9196799; doi:10.1080/15592324.2022.2084277)
Supplement: Supplemental Material [file KPSB_A_2084277_SM1927.docx]

**Supplementary Table 1. PCR primers used in this study**

| Name | Locus | Sequence (5’→3’) | A.T.^a)^ | Experiment |
| --- | --- | --- | --- | --- |
| *BEH2* | AT4G36780 | AGATTCAGAGCTCTTCAATGGCCGC | 67 | sqRT-PCR |
|  |  | ATTTACCGGTTTTTCAGCATCTGGC |  |  |
| *DWF4* | AT3G50660 | TACCTCTTCTTCTTCTCCCATCGC | 55 |  |
|  |  | CGAGAAACCCTAATAGGCAAACCG |  |  |
| *ACT2* | AT3G18780 | TTCCGCTCTTTCTTTCCAAGCTCA | 50 |  |
|  |  | AAGAGGCATCAATTCGATCACTCA |  |  |
| *GUS* | - | GTGCCAGGCAGTTTTAACGA | 59 |  |
|  |  | ATGCGTCACCACGGTGATAT |  |  |
| *BEH2* promoter | - | CCTCTAGATGATTCTTTCTGGATAAGTC | 55 | Construction of *BEH2::GUS* |
|  |  | CCGGATCCAAGTCTTTGTCAGACTGGCGC |  |  |
| *BEH2* CDS | - | GGACGTCGACCAGAGATTCAGAGCTCTTCAATG | 60 | Construction of *35S::BEH2:GFP* |
|  |  | GCCGCCATGGGGCATCTGGCTTTAGTGCCACCGA |  |  |
| *BZR1* CDS | - | GGACGGTACCGGTTGTTGGTTTTCCCGATG | 53 | Construction of *35S::BZR1:GFP* |
|  |  | GCCGGGATCCACCACGAGCCTTCCCATTTCCA |  |  |

^a)^ Annealing temperature (°C)


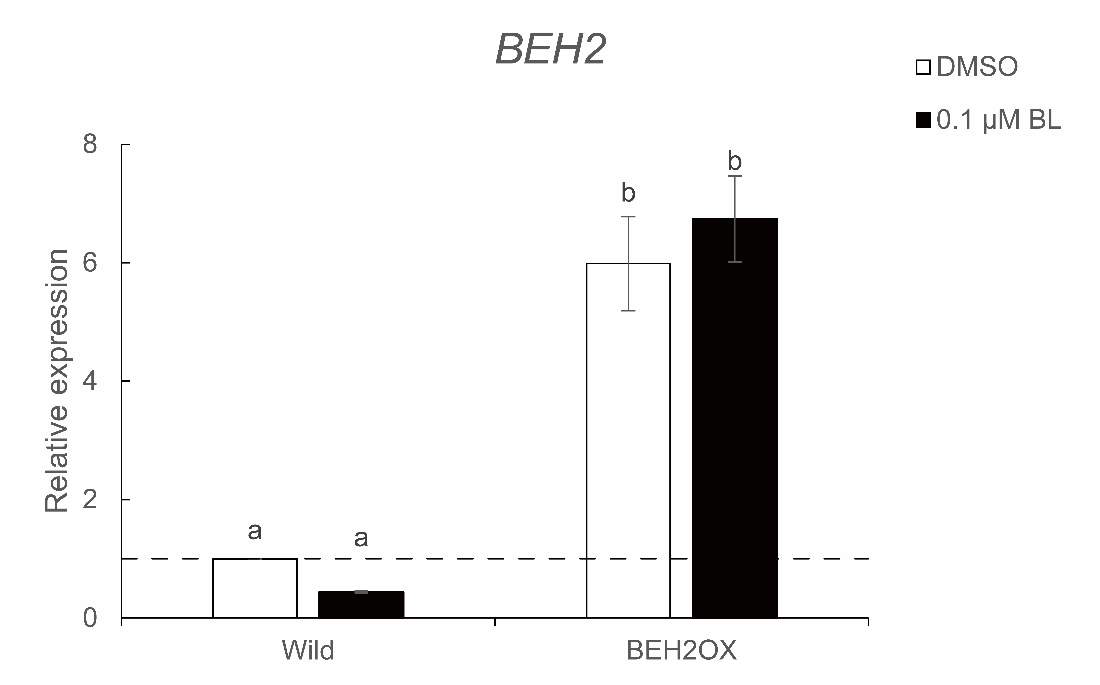


**Supplementary Figure 1. *BEH2*** **expression in its overexpressing plant**

14-day-old *Arabidopsis* WT (Col-0) and *BEH2* overexpressing plants (Col-0 background) were treated with either BL or DMSO for 4 h and subjected to quantitative RT-PCR for evaluating *BEH2* mRNA level. Two sets of primers (5’-CGATGGCACCACTTATCGCA-3’ / 5’-GTGGTACGAAGGTGCAGGAC-3’ and 5’-GCCATCCAAGCTGTTCTCTC-3’ / 5’-CCCTCGTAGATTGGCACAGT-3’) were used to amplify mRNAs of *BEH2* and *ACT2* as a control, respectively. Presentation styles in the graphs follow those in Figure 1. Statistical analysis was performed by ANOVA with Tukey’s test (p < 0.01).


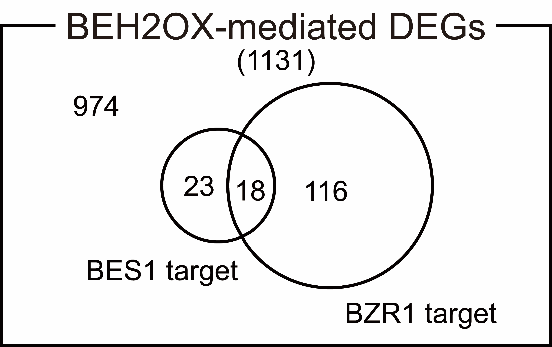


**Supplementary Figure 2. Overlap between BEH2OX-mediated DEGs and BES1/BZR1 target genes**

Venn diagram showing the number of BES1- (Yu et al., 2011) and BZR1-targets (Sun et al., 2010) included in a pool of BEH2OX-mediated DEGs (1131).


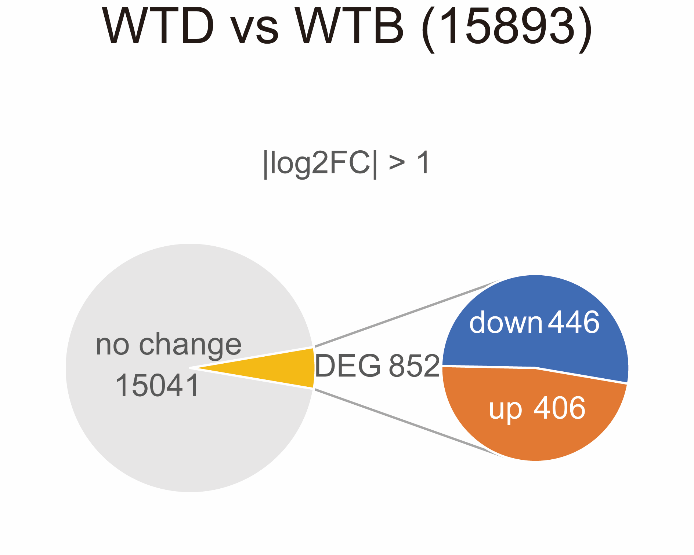


**Supplementary Figure 3. Transcriptomic profile of BL-treated WT seedling**s

Pie charts indicating the proportion of DEGs with |log2FC| >1 among the total genes (15893) used for comparison between WT plants grown in the absence and presence of BL (WTD vs WTB).


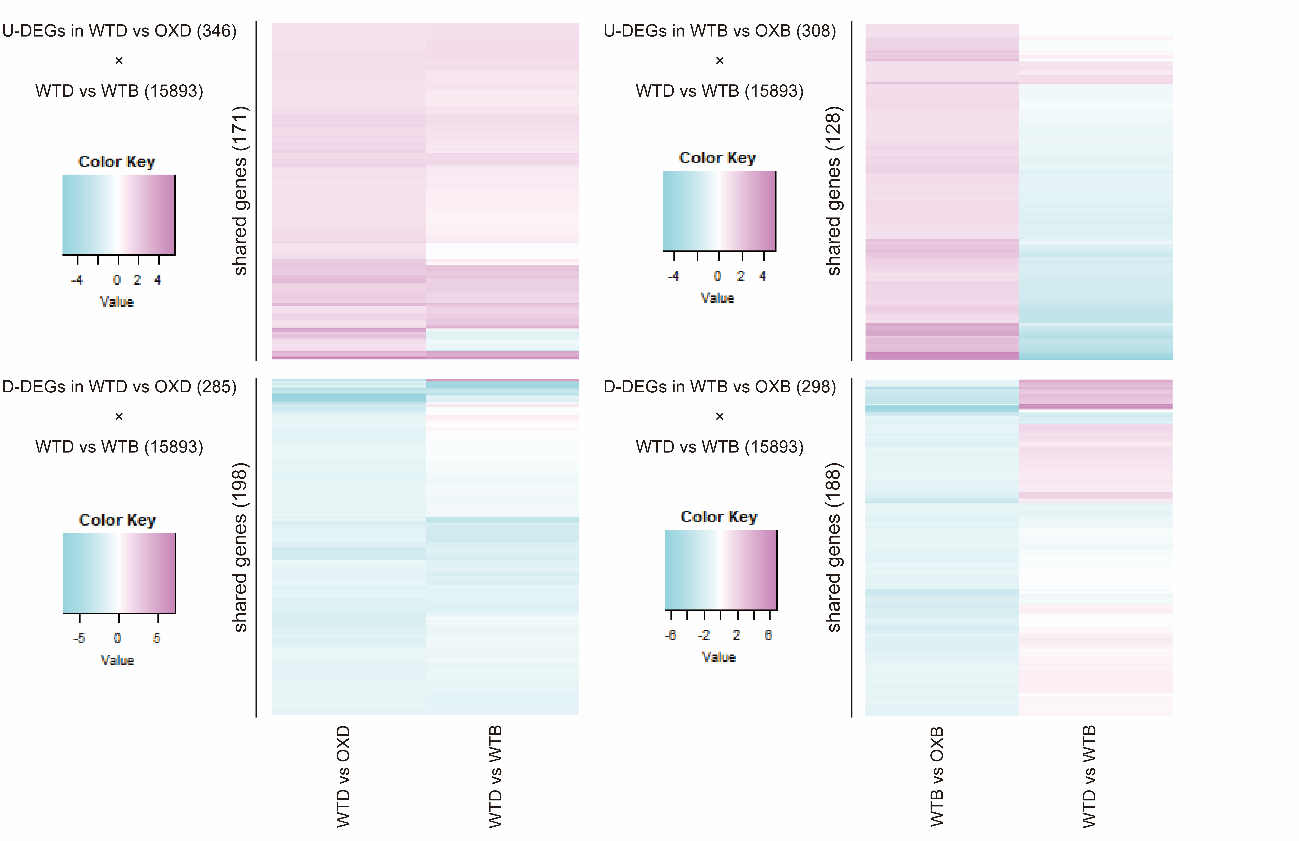


**Supplementary Figure 4. BR responsiveness of BEH2OX-mediated DEGs in WT**

The shared genes (171, 198, 128, and 188 for each pool) found both in four pools of BEH2OX-mediated DEGs (U-DEGs and D-DEGs in WTD vs OXD; U-DEGs and D-DEGs in WTB vs OXB) and in 15893 genes used for comparison between WTD and WTB were aligned to produce the heatmap using logFC value.


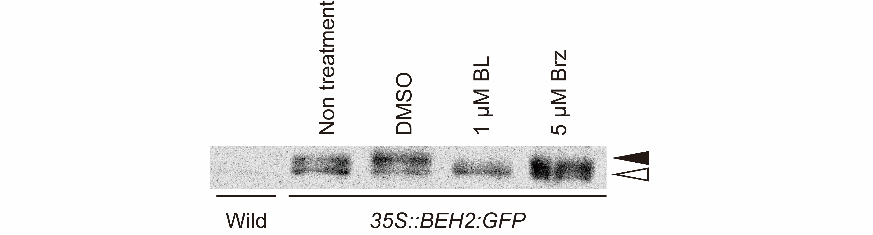


**Supplementary Figure 5. Immunoblot of BEH2:GFP fusion protein**

Immunoblot using anti-GFP antibody. 14-day-old *35S::BEH2:GFP* plants were cultured for 1 day in 1/2 MS liquid medium containing either BL or Brz and then subjected to immunoblot analysis. Closed- and open arrowhead showing the phosphorylated and dephosphorylated BEH2:GFP, respectively.
